# Outcome of Ligation without Revascularization in Pseudoaneurysms of Peripheral Arteries in Intravenous Drug Users

**DOI:** 10.31729/jnma.4472

**Published:** 2019-06-30

**Authors:** Lokesh Shekher Jaiswal, Narendra Pandit, Shailesh Adhikary

**Affiliations:** 1 Department of Surgery, BP Koirala Institute of Health Sciences, Dharan, Sunsari, Nepal

**Keywords:** *intravenous ligation*, *peripheral artery*, *pseudoaneurysm*, *substance abuse*

## Abstract

**Introduction:**

Management of pseudoaneurysms in intravenous drug users is complex and challenging due to an associated infection and unavailability of autologous vein grafts. Here we observe the outcomes of ligation and local debridement as a primary modality of treatment in this subset of patients with pseudoaneurysms.

**Methods:**

This is a descriptive cross sectional study of 15 patients over a period of 4 years who presented with pseudoaneurysm of peripheral artery from intravenous drug use. In this study, we describe the presentations and management outcomes in 15 patients with peripheral arterial pseudoaneurysmfrom IV drug use.

**Results:**

The most common site involved was common femoral artery among 12 (80%) patients followed by superficial femoral artery among 8 (13.3%) patients and external iliac artery in 1 (6.7%) patient. Twelve (80%) patients were having signs of infection. All patients underwent surgical intervention which comprised of excision of pseudoaneurysm and ligation of artery without revascularization among 12 (80%) patients and with revascularization with autologous venous graft among 3 (20%) patients. There was no mortality or a major bleeding requiring re-exploration. None of the patients developed limb ischemia necessitating amputation. One patient with femoral artery ligation without revascularization at one year of follow up is having claudication on brisk walking. There was one saphenous vein graft thrombosis in immediate postoperative period.

**Conclusions:**

With the use of ligation without revascularization technique, there was no mortality or major bleeding requiring re-exploration. None of the patients developed limb ischemia necessitating amputation so this treatment modality seems promising in treatment of pseudoaneurysms in intravenous drug users.

## INTRODUCTION

Pseudoaneurysm is a localized pulsatile hematomahaving persistent connection with the vessel lumen.^1^ Vascular interventions and intravenous (IV) drug use are the most common causes.^2^ There is annual increase in IV drug users in Nepal, thus increasing the number of patients with pseudoaneurysm.^3^

The management of pseudoaneurysm varies from open surgical repair to minimal invasive interventions.^4,5^ The conventional surgical treatment is excision and debridement of pseudoaneurysm followed by routine revascularisation.^6^ In IV drug user, surgery is the mainstay of management due to associated infection and skin necrosis. In addition, there is limited availability of autologous vein graft and revascularization is associated with high incidence of complications in IV drug users.^7^ Therefore, many centers have adopted the strategy of no revascularisation or selective revascularisation.^7,8^

In this study, we describe the presentation and management outcomes of 15 consecutive patients presenting with peripheral artery pseudoaneurysm from IV drug abuse at our centre in eastern Nepal.

## METHODS

The retrospective chart review of the patients managed for peripheral vascular pseudoaneurysm from October 2014 to January 2019 at BP Koirala Institute of Health Sciences (BPKIHS) of Nepal was done. Convenient sampling was done. The study protocol was approved by Institutional Review Committee of BPKIHS (IRC-1179/17).


$$\text{n}={\text{Z}}^{2}\times (\text{pq)/}{\text{d}}^{2}$$

where,

n = sample size
p = prevalence, 5% (educated guess)
q = 1-p
d = margin of error, 11%
Z = 1.96 at 95% CI

All patients with pseudoaneurysm of peripheral arteries due to IV drug abuse were included in this study. Patients with pseudoaneurysm due to other causes were excluded. We retrospectively evaluated inpatient and anesthesia records for presentation, surgical intervention performed and their early outcomes of 15 patients. All patients followed the same treatment protocol. The diagnosis was confirmed in all patients by ultrasound followed by CT angiogram and when required.

The surgical procedure included excision of pseudoaneurysm and debridement of necrotic tissue and pus collections followed by ligation of artery with or without distal revascularization.

After dissection of a proximal healthy artery, a clamp was applied temporarily and oxygen saturation of affected limb was monitored with pulse oximetry. When oxygen saturation was more than 90%, ligation of artery along with debridement of infected and necrotic tissue was formed without revascularization. Revascularization with autologous vein was performed when saturation was less than 90% and/or when the limb was cold with delayed capillary refill time. Processing of data was performed using the Microsoft Excel 2007. Descriptive statistics was used and data are presented as mean (standard deviation) for continuous variables or median and ranges as appropriate. Total counts and percentages are reported for categorical variables.

## RESULTS

A total of 15 patients were operated for pseudoaneurysms of peripheral arteries due to IV drug abuse over the period of four years along with patient characteristics (Table 1).

**Table 1.** Summary of Patients Characteristics.

Variable		n (%)
Age (mean, Year)		28
Sex		
Male		
		15 (100%)
Comorbidities		
	Chronic Hepatitis C virus	
	infection	2 (53.3%)
	Hepatitis B	1 (40%)
	DVT	6 (33.3%)
Symptoms/history		
	Pain	
	Pulsatile Mass	15 (100%)
	Palpable Thrill	13 (86.7%)
	Fever	13 (86.7%)
	Bleeding	11 (73.3%)
	Skin discoloration/	7 (46.7%)
	ulceration	6 (40%)
Intervention		
	Ligation	12 (80%)
	Venous graft bypass	3 (20%)
Artery Involved		
	Common Femoral	12 (80%)
	Superficial Femoral	2 (13.3%)
	External Iliac	1 (6.7%)

The average duration of symptoms was 3 months and the average duration of IV drug use was 2.7 years ranging from less than a year to 5 years. The most common presenting complaint was a pulsatile swelling followed by pain, fever and bleeding. Groin was the most site of involvement. Five (33.3%) patients were having frank necrosis and loss skin of thigh.

Five (33.3%) patients were having associated deep vein thrombosis (DVT). Duplex ultrasonography was done in all patients to confirm the diagnosis. CT angiogram was done only in five (33.3%) patients. The most common artery involved was common femoral artery among 12 (80%) patients followed by superficial femoral arteryamong 2 (13.3%) patients and external iliac artery in 1 (6.7%) patients.

The operative technique for proximal control of artery varied in relation to local wound conditions. Proximal control of external iliac artery was done via suprainguinal retroperitoneal approach in nine (60%) patients and midline intraperitoneal approach in six patients (40%). Excision of pseudoaneurysm and debridement of necrotic tissue with ligation of proximal and distal ends without distal revascularization was done in 12 (80%) and distal revascularization with autologous venous graft was done in 3 (20%) patients (Table 2).

**Table 2.** Postoperative outcome.

Outcome Parameter	Ligation only n = 12 (80%)	Ligation with revascularization n = 3 (20%)
Mortality	Nil	Nil
Limb gangrene	Nil	Nil
Amputation	Nil	Nil
Re-exploration for hemorrhage	Nil	Nil
Graft thrombosis	NA	1
Claudication	1 (8%)	Nil

There was no mortality or re-exploration for hemorrhage. None of the patients without distal revascularization developed limb ischemia or amputation. One patient out of three with saphenous vein bypass developed graft thrombosis without limb gangrene and claudication in postoperative follow up. Seroma developed in 3 (20%) patients following surgery which was managed conservatively.

In 10 (66.7%) patients, bacteria were isolated in culture of necrotic material and hematoma. The predominant organism isolated was staphylococcus aureus in 4 (26.7%) patients andrest of the pseudoaneurysm showed mixed bacterial infection comprising of aerobic and anaerobic bacteria. All patients received intravenous antibiotics during hospital stay followed by oral antibiotics for one more week after discharge. Postoperatively all patients underwent counseling for de-addiction.

All patients are in regular follow up with mean duration of 22 months. One patient without distal revascularization is having claudication on brisk walking.

## DISCUSSION

Peripheral pseudo aneurysm scan be managed with either open surgical repair or other minimal invasive procedure like ultrasound guided compression/thrombin injection and covered stent graft implantation. Indications for the surgical repair of pseudoaneurysms include rapid expansion (especially in an unstable patient), distal ischemia, infection, neuropathy, overlying soft tissue or skin ischemia, impending compartment syndrome, and the failure of percutaneous treatment. The operative technique and choice of vascular reconstruction varies with the etiology and the presentation of the patient.^4,5^

The treatment of pseudoaneurysms in IV drug user is mainly surgical as they are mostly infected due to repeated puncture of artery with unsterile needle. Untreated infected pseudoaneurysm can lead to sepsis, hemorrhage, limb loss and death. In our study, 80% patients were showing clinical signs of infection in pseudoaneurysm, thus excluding the need of minimal invasive procedure. There is no consensus yet on type of revascularization after ligation of artery in infected pseudoaneurysms of IV drug users. The options include revascularization with autologous vein or prosthetic graft, selective delayed revascularisation and ligation of artery without revascularisation. In our review, all patients had pseudoaneurysm at the groin region and required debridement of abscess/hematoma and necrotic material followed by ligation of the artery. None of the patients undergoing simple ligation developed limb ischemia, thus establishing its safety at a centre with limited resources like ours. The use of conduit in infected pseudoaneurysms can be problematic due to graft thrombosis, hemorrhage and progression to septicemia.^9^

Studies have shown ligation of artery without revascularisation is associated with high incidence of lower extremity ischemia and limb loss especially in traumatic condition and triple ligation, thus preventing its widespread use.^10,11^ The same principle cannot be utilized in IV drug user as they are found to have better collaterals. High incidence of amputation has been reported in studies where intraoperative evidence of collaterals in the form of saturation and pedal doppler signal were not searched.^12,13^ Studies where ligation was done with intraoperative evidence of collateral (by adequate doppler signal in distal pedal artery, adequate capillary refill time and saturation on pulse oximetry) during intraoperative test occlusion, limb ischemia was as low as 0 to 8%.^9,14,15^ There are several advantages of ligation alone. The conduit issue is avoided, the operative time in septicemia is reduced, and the complications are minimal as has been seen in our study. The incidence of prosthetic graft infection even when placed deep in the extra anatomic location is high. We routinely test clamp the proximal healthy artery and measure the distal oxygen saturation with pulse oximetry. The culprit artery is ligated without distal revascularization if saturation is more than 90%, and the infected necrotic tissue of pseudoaneurysm with pus pockets is debrided. Postoperatively, we observe and monitor patients for any evidence of limb ischemia.

Only ligation and excision of pseudoaneurysm predisposes to late complications like claudication especially if it involves bifurcation necessitating triple ligation.^12^ The option for these situations can be extra-anatomic bypass or delayed revascularization, however the risk conduit infection and hemorrhage still persists.^6^ Another potential risk of using superficial conduit in IV drug abuser is continued use of IV drug by some, thus leading to risk of recurrence and bleeding if on anticoagulation for deep vein thrombosis. One patient (8%) in our studyhad claudication on brisk walkings.

## CONCLUSIONS

IV drug abuse is still a common cause of pseudoaneurysm and it usually presents with infected condition. Ligation and evacuation of infected tissues without revascularization is a technically less challenging procedure andseems promising in treatment of pseudoaneurysm in intravenous drug users.

## Conflict of Interest

**None.**
